# Early blood pressure lowering therapy is associated with good functional outcome in patients with intracerebral hemorrhage

**DOI:** 10.1186/s12883-024-03561-y

**Published:** 2024-02-14

**Authors:** Xinni Lv, Xueyun Liu, Zicheng Hu, Lan Deng, Zuoqiao Li, Jing Cheng, Mingjun Pu, Qi Li

**Affiliations:** 1 Department of Neurology, The First Affiliated Hospital of Chongqing Medical University, Chongqing, 400016 China; 2 Department of Neurology, The Second Affiliated Hospital of Anhui Medical University, Hefei, China; 3 Department of Neurology, People’s Hospital of Chongqing Hechuan (PHHC), Chongqing, China; 4 Department of Neurology and Neurosurgery, The Third Affiliated Hospital of Chongqing Medical University, Chongqing, China

**Keywords:** Intracerebral hemorrhage, Outcome, Blood Pressure, Treatment

## Abstract

**Background:**

The implementation of a care bundle might improve functional outcome for patients with intracerebral hemorrhage (ICH). However, the impact of anti-hypertensive treatment on ICH outcomes remains uncertain. Our objective is to examine whether early blood pressure (BP) lowering therapy within first 12 h is associated with good outcome in ICH patients.

**Methods:**

We included acute ICH patients who had baseline computed tomography (CT) scans within 6 h after onset of symptoms between October 2013 and December 2021. Early BP reduction was defined as use of anti-hypertensive agents within 12 h after onset of symptom. The clinical characteristics were compared between patients who received early BP lowering therapy and those without. The associations between early BP lowering and good outcome and functional independence at 3 months were assessed by using multivariable logistic regression analyses.

**Results:**

A total of 377 patients were finally included in this study for outcome analysis. Of those, 212 patients received early BP reduction within 12 h after ICH. A total of 251 (66.6%) patients had good outcome. After adjustment for age, admission systolic BP, admission GCS score, baseline hematoma volume, hematoma expansion, and presence of intraventricular hemorrhage, early BP lowering therapy was associated with functional independence (adjusted odd ratio:1.72, 95% confidence interval:1.03–2.87; *P* = 0.039) and good outcome (adjusted odd ratio: 2.02, 95% confidence interval:1.08–3.76; *P* = 0.027).

**Conclusions:**

In ICH patients presenting within 6 h after symptom onset, early BP reduction within first 12 h is associated with good outcome and functional independence when compared to those who do not undergo such early intervention. Implementation of quality measures to ensure early BP reduction is crucial for management of ICH.

## Introduction

Spontaneous intracerebral haemorrhage (ICH) is the most lethal form of stroke, affecting ≈2–4 million people worldwide each year [1–3]. It is a devastating neurological condition associated with high morbidity and mortality, as only 20%-39% of patients are expected to be functionally independent at 6 months [3]. An acute hypertensive response is frequently observed in patients with acute ICH in the first few hours after onset and is associated with poor functional outcome following ICH [4–7]. Therefore, early reduction of elevated blood pressure (BP) after ICH seems to be a promising therapeutic strategy that could potentially attenuate hematoma expansion and reduce the risks of recurrent ICH and poor outcome.

Two large prospective, multi-centre, randomized controlled trials, the second Intensive Blood Pressure Reduction in Acute Cerebral Hemorrhage Trial (INTERACT2) and Antihypertensive Treatment of Acute Cerebral Hemorrhage Trial (ATACH-2), investigated early intensive BP lowering compared to standard BP management [8, 9]. The INTERACT-2 trial, involving 2829 patients, showed that there were no clear differences in rates of death or severe disability at 90 days between the intensive BP lowering group and the control group [8]. Similarly, the subsequent large trial, the ATACH2 trial, reported no benefit of the intensive BP lowering treatment [9]. Of note, intensive BP lowering was not significantly associated with improved primary outcome in the INTERACT-2 and ATACH-2 trials.

Recently, The third Intensive Care Bundle with Blood Pressure Reduction in Acute Cerebral Haemorrhage Trial (INTERACT3) showed that the implementation of a care bundle can improve functional outcome for patients with ICH [10]. The mean difference of achieved blood pressure was 7.3 mmHg at 1 h and 3.6 mmHg at 24 h between treatment and control groups in INTERACT3. Therefore, the impact of anti-hypertensive treatment on ICH outcomes and the optimal timing of BP reduction remains uncertain. In acute ischemic stroke patients, early BP modulation was not associated with improved functional outcome [11].

While several studies have compared the impact of intensive versus standard blood pressure reduction on hematoma expansion and functional outcomes in individuals with ICH, there has been limited exploration of patients who underwent early blood pressure reduction compared to those who did not [8–10]. In order to evaluate the efficacy of early BP lowering therapy and its impact on functional outcome, we compared the functional outcomes of patients who received early BP reduction to those without. Since most hematoma growth occurs within 12 h after symptom onset [12], we operationally defined early BP reduction as the use of anti-hypertensive agents within 12 h after symptom onset. We hypothesized that initiating BP-lowering therapy within this time frame would be associated with good functional outcome in ICH patients.

## Methods

### Ethical standards

The study protocol was approved by the Ethics Committee of the First Affiliated Hospital of Chongqing Medical University. Informed consent was waived due to the retrospective nature of this study (the Ethics Committee of the First Affiliated Hospital of Chongqing Medical University). All methods were performed in accordance with the relevant guidelines and regulations or declaration of Helsinki.

### Study design

We analyzed consecutive patients from an ongoing prospective cohort of primary ICH who were hospitalized in the First Affiliated Hospital of Chongqing Medical University between October 2013 and December 2021. Patients aged > 18 years were enrolled in our study if they performed baseline computed tomography (CT) scans within 6 h after ICH onset. Follow-up CT scan was performed within 36 h after the baseline CT scan. Patients were grouped according to whether they received anti-hypertensive medications during the first 12 h after onset of symptom. Patients were excluded, if they had: (1) secondary ICH due to structural lesions (e.g., intracranial aneurysm, moyamoya disease, and arteriovenous malformation), anticoagulant-associated bleeding, and hemorrhagic transformation from acute ischemic infarction; (2) trauma and tumor; (3) primary intraventricular hemorrhage. Patients who underwent surgical intervention before the follow-up CT scans or without follow-up imaging data were also excluded. Patients who are admitted to the hospital with a systolic blood pressure (SBP) below 150 mmHg were also excluded. The demographic and clinical variables, including age, sex, admission BP, time from onset to admission CT scan, stroke severity were prospectively collected. Laboratory test results, anti-hypertensive medication use, length of hospital stay, and neurological or systemic complications were recorded by the research staff.

### Imaging analysis and outcome assessment

The admission and follow-up CT scans images were saved as Digital Imaging and Communications in Medicine format for further review. All the images were independently reviewed by two trained neurologists who were blinded to clinical and outcome data. Hematoma volume was measured by using ABC/2 method. Hematoma location was classified as deep (basal ganglia, thalamus) and non-deep. Hematoma growth was defined as an absolute hematoma growth > 6 mL from baseline to follow-up CT scan [13]. Functional outcome was assessed by using the modified Rankin Scale (mRS) at 3 months by telephone interviews. We defined functional independence at 3 months as an mRS score of 0–1, and good outcome as an mRS score of 0–3.

### Statistical analysis

Categorical variables were presented as count (%). Continuous data are summarized either as median and inter-quartile ranges or mean and standard deviations (SD). The baseline characteristics, including demographic, clinical and radiological variables, were compared between patients with hematoma growth and those without by using Fisher’s exact test, or Student’s t test, or Mann–Whitney U test, or chi-squared test as appropriate. The effect of early anti-hypertensive drug use on functional independence and good outcome was assessed by using multivariable logistic regression analyses. Multivariate logistic regression analyses were performed by including covariates with *p* < 0.1 in univariable logistic regression. We further performed the multivariate linear regression analysis. The multi-collinearity among variables was evaluated on the basis of variance inflation factor (VIF). And a predictor of VIF > 5 was considered indicative of collinearity. The statistical significance level was set at *p* < 0.05. All statistical analyses were performed by using the SPSS software(version 25.0; IBM Corporation, Armonk, NY, United States).

## Results

**Fig. 1:**
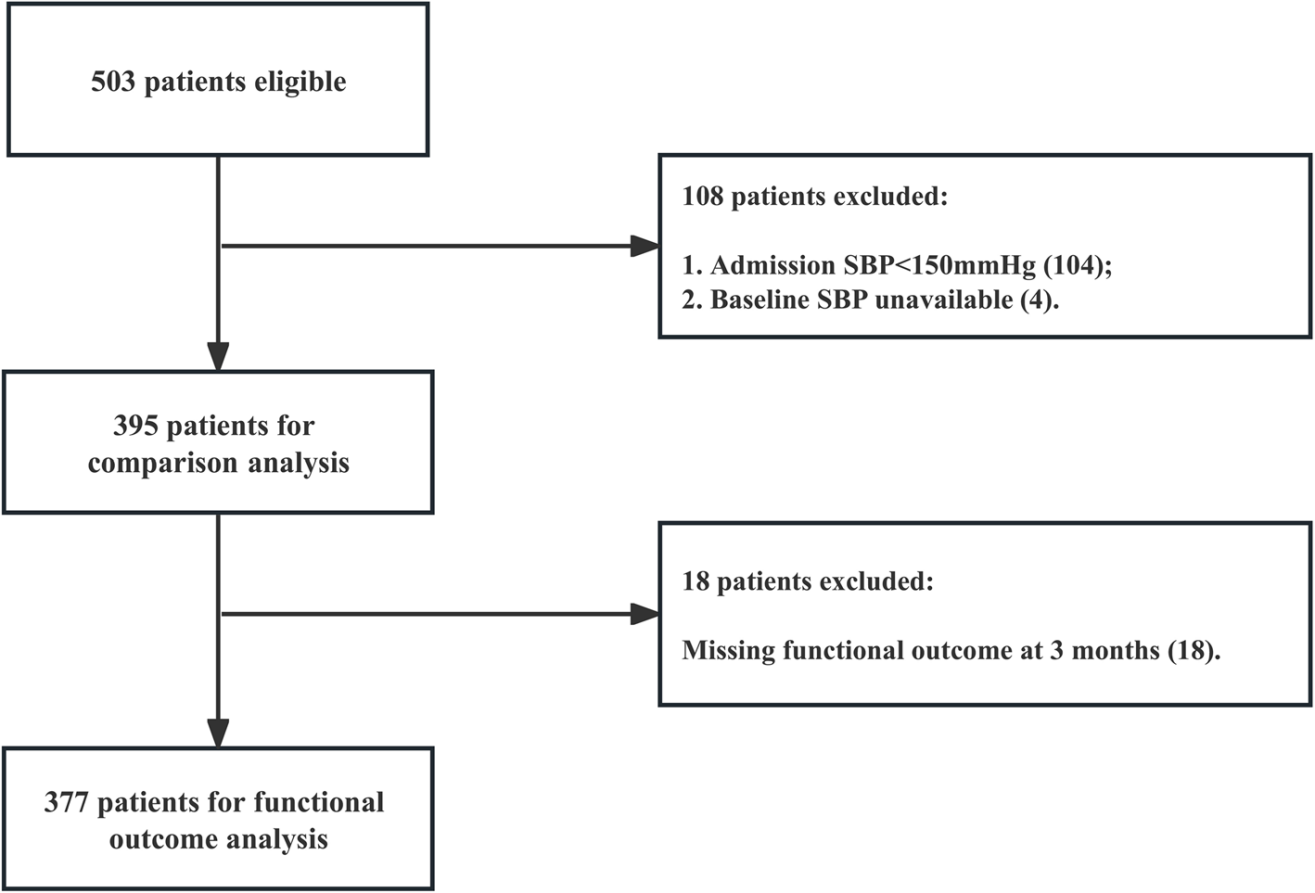
Flow chart showing the selection of eligible patients

A total of 503 patients met the inclusion criteria during the study period. Of these, 377 patients were included in outcome analysis. The flowchart is illustrated in Fig. 1. A total of 126 patients were excluded owing to missing data on systolic BP (*n* = 4), patients with BP < 150 mmHg at admission (*n* = 104), and loss to follow-up (*n* = 18). The mean age of included patients was 59.8 ± 13.4 years. The median time from ICH onset to initial CT scan was 2.1 h (interquartile range, 1.3–3.4 h).The hematomas were located in basal ganglia (*n* = 241), thalamus (*n* = 63), cerebral lobes (*n* = 49), brainstem (*n* = 26) and cerebellum (*n* = 16). The median baseline BP was 179 mmHg (interquartile range, 165-198 mmHg). Of 377 patients, 212 (56.2%) received anti-hypertensive medication within 12 h. The median time from onset to administration of first anti-hypertensive agent was 4.0 h (inter-quartile range, 2.7–5.7 h) in treatment group.

Comparison of baseline demographics, clinical and radiological characteristics between patients with and without early BP reduction therapy were shown in Table 1. Patients who received early BP lowering treatment were more likely to have history of hypertension (68.31% vs 81.13%, *P* = 0.003). There were no significant differences between the two groups in terms of admission SBP (*P *= 0.216), admission GCS score (*P* = 0.532), NIHSS score (*P* = 0.728), baseline hematoma volume (*P* = 0.601), or time from onset to CT (*P* = 0.526).

**Table 1:** Comparison of baseline, clinical, and radiological characteristics between patients with and without receiving early BP reduction

Variables	Without early BP lowering treatment (*n* = 183)	Early BP lowering treatment (*n* = 212)	*P* value
**Demographics**			
Mean age, year (SD)	59.99 (12.87)	59.56 (13.79)	0.750
Sex, male, n(%)	121 (66.12)	147 (69.34)	0.495
**Medical history**			
Alcohol consumption, n (%)	62 (33.88)	82 (38.68)	0.212
Smoking, n (%)	79 (43.17)	106 (50.00)	0.175
History of Hypertension, n (%)	125 (68.31)	172 (81.13)	0.003
History of diabetes, n (%)	25 (13.66)	35 (16.51)	0.461
Previous ischemic stroke, n (%)	16 (8.74)	14 (6.60)	0.438
Previous hemorrhagic stroke, n (%)	14 (7.65)	20 (9.43)	0.510
**Clinical features**			
Admission SBP, mmHg (SD)	181.93 (23.34)	184.81 (22.74)	0.216
Median admission GCS score (IQR)	14 (10–15)	14 (12–15)	0.532
Median admission NIHSS score (IQR)	10 (6–17)	11 (5–16)	0.728
Median hematoma volume, ml (IQR)	11.47 (6.41–22.15)	11.30 (6.03–20.68)	0.601
Hematoma expansion, n (%)	31 (16.94)	41 (19.34)	0.538
Median time from onset to imaging, h (IQR)	2.12 (1.31–3.25)	2.00 (1.26–3.60)	0.526
Median time from onset to treatment, h (IQR)	-	4.03(2.7–5.67)	-
IVH at baseline CT, n (%)	63(34.43)	74 (34.91)	0.920
Deep location, n (%)	148 (80.87)	156 (73.58)	0.102
**Laboratory values**			
White blood cell count, 10^9^/L(IQR)	9.49(7.32–11.40)	9.97(7.48–12.73)	0.151
Hemoglobin, g/dL(IQR)	141.0(132.0–152.0)	143.0(133.0–155.0)	0.282
Platelets,10^9^/L(IQR)	179.0(143.5–221.0)	181.0(140.0–216.3)	0.540
Serum creatinine,μmol/L(IQR)	66.0(56.0–81.0)	71.0(57.0–86.0)	0.060
Blood glucose level, mmol/L	6.7(5.6–8.3)	7.2(5.9–9.2)	0.032

*SD* standard deviation, *SBP* systolic blood pressure, *GCS* Glasgow Coma Scale, *NIHSS* National Institute of health stroke scale, *IQR* inter-quartile range, *IVH:* intraventricular hemorrhage

A total of 126 (33.4%) patients in our cohort had poor functional outcome (mRS 4–6) at 3 months. The baseline demographics, clinical, and imaging characteristic between patients with and without poor functional outcome were shown in Table 2. Patients with poor outcome had older age (63.4 vs 58.1, *P* < 0.001), higher admission SBP (188.7 vs 181.1, *P* = 0.002), lower admission GCS score (14 vs 11, *P* < 0.001), higher NIHSS score (17 vs 8, *P* < 0.001), larger baseline hematoma volume (17.6 vs 9.4, *P* < 0.001), higher white blood cell count (10.47 vs 9.35, *P* = 0.001), higher serum creatinine (72.0 vs 68.0, *P* = 0.015) and blood glucose level (8.1 vs 6.7, *P* < 0.001) than those without.

**Table 2:** Comparison of baseline demographic, clinical, and radiological characteristics between patients with and without good outcome

Variables	Good outcome (*n* = 251)	Poor outcome (*n* = 126)	*P* value
**Demographics**			
Mean age, year (SD)	58.10 (13.03)	63.38 (13.71)	< 0.001
Sex, male, n(%)	174 (69.32)	84 (66.67)	0.601
**Medical history**			
Alcohol consumption, n (%)	100 (39.84)	40 (31.75)	0.124
Smoking, n (%)	122 (48.61)	57 (45.24)	0.537
History of Hypertension, n (%)	181 (72.11)	102 (80.96)	0.061
History of diabetes, n (%)	30 (11.95)	29 (23.02)	0.005
Previous ischemic stroke, n (%)	15 (5.98)	13 (10.32)	0.129
Previous hemorrhagic stroke, n (%)	13 (5.18)	18 (14.29)	0.003
**Clinical features**			
Admission SBP, mmHg (SD)	181.07 (21.08)	188.74 (26.15)	0.002
Median admission GCS score (IQR)	14 (13–15)	11 (7–14)	< 0.001
Median admission NIHSS score (IQR)	8 (4–12)	17 (12–26)	< 0.001
Median hematoma volume, ml (IQR)	9.43 (4.78–17.50)	17.59 (9.51–32.33)	< 0.001
Hematoma expansion, n (%)	32 (12.75)	39 (30.95)	< 0.001
Median time from onset to imaging, h (IQR)	2.18 (1.27–3.55)	2.00 (1.00–3.18)	0.174
Median time from onset to treatment, h (IQR)	6.08 (3.52–25.40)	5.68 (3.27–32.75)	0.840
Early BP reduction within 12 h, n (%)	142 (56.57)	60 (47.62)	0.100
IVH at baseline CT, n (%)	55 (21.91)	74 (58.73)	< 0.001
Deep location, n (%)	191 (76.10)	100 (79.37)	0.516
**Laboratory values**			
White blood cell count, 10^9^/L(IQR)	9.35 (7.25–11.90)	10.47 (8.08–13.04)	0.001
Hemoglobin, g/dL(IQR)	142.0 (134.0–156.0)	141.5 (130.0–149.3)	0.061
Platelets,10^9^/L(IQR)	183.0 (147.5–222.5)	173.0 (133.0–208.0)	0.160
Serum creatinine,μmol/L(IQR)	68.0 (56.0–80.8)	72.0 (60.0–90.0)	0.015
Blood glucose level, mmol/L	6.7 (5.6–7.9)	8.1 (6.3–11.2)	< 0.001

*SD* standard deviation, *SBP* systolic blood pressure, *GCS* Glasgow Coma Scale, *NIHSS* National Institute of health stroke scale, *IQR* inter-quartile range, *IVH* intraventricular hemorrhage

A total of 138 (36.6%) patients in our cohort had functional independence (mRS 0–1) at 3 months. In multivariable analysis, early BP lowering therapy was associated with functional independence (adjusted OR 1.72, 95% CI 1.03–2.87; *P* = 0.039) after adjusting for age, admission SBP, baseline GCS, previous hypertension, log-transformed hematoma volume, presence or absence of IVH, and hematoma expansion (Table 3). Multivariate logistic regression analysis was also performed to predict good outcome after ICH at 3 months (Table 4). After controlling for age, admission SBP, baseline GCS score, log-transformed hematoma volume, presence or absence of IVH, hematoma expansion, previous hypertension, white blood cell count, serum creatinine, blood glucose level, early BP lowering treatment was associated with good outcome (adjusted OR, 2.02, 95% CI 1.08–3.76; *P* = 0.027) (Table 4).

**Table 3:** Factors associated with functional independence (mRS 0–1)

**Variables**	**Multivariable analysis**^a^	
	**OR (95% CI)**	***P* value**
age	0.97 (0.95–0.99)	0.001
Admission SBP	0.98 (0.97–0.99)	0.002
admission GCS	1.35 (1.18–1.54)	< 0.001
Hematoma volume	0.53 (0.30–0.95)	0.034
Baseline IVH	0.41 (0.23–0.74)	0.003
Hematoma expansion	0.16 (0.06–0.44)	< 0.001
Early BP reduction within 12 h	1.72 (1.03–2.87)	0.039

*mRS* modified Rankin Scale, *OR* odds ratio, *CI* confidence interval, *SBP* systolic blood pressure, *BP* blood pressure, *GCS* Glasgow Coma Scale, *IVH* intraventricular hemorrhage

^a^Adjusted for age, admission SBP, presence of IVH, log-transformed hematoma volume, previous hypertension, admission GCS, hematoma expansion and early BP reduction within 12 h

**Table 4:** Factors associated with good outcome (mRS 0–3)

**Variables**	**Multivariable analysis**^a^	
	**OR (95% CI)**	***P* value**
Age	0.96 (0.94–0.99)	0.004
Admission SBP	0.99 (0.97–0.99)	0.048
admission GCS	1.25 (1.11–1.39)	< 0.001
Hematoma volume	0.36 (0.16–0.81)	0.014
Baseline IVH	0.26 (0.14–0.50)	< 0.001
Hematoma expansion	0.16 (0.07–0.35)	< 0.001
Early BP reduction within 12 h	2.02 (1.08–3.76)	0.027

*mRS* modified Rankin Scale, *OR* odds ratio, *CI* confidence interval, *SBP* systolic blood pressure, *BP* blood pressure, *GCS* Glasgow Coma Scale, *IVH* intraventricular hemorrhage

^a^Adjusted for age, admission SBP, presence of IVH, log-transformed hematoma volume, previous hypertension, admission GCS, hematoma expansion, white blood cell count, serum creatinine, blood glucose level and early BP reduction within 12h

## Discussion

In our study, we evaluated whether those patients treated within 12 h of symptom onset benefited from early BP lowering treatment. We found that lower admission SBP is associated with good outcome and early anti-hypertensive drug use is associated with good outcome and functional independence in patients with ICH.

Hypertensive response occurs in approximately 50–80% of patients at time of admission after ICH [4, 6]. In acute ischemic stroke, early BP modulation was not associated with improved functional outcome [11]. In theory, early BP reduction may diminish arterial perfusion surrounding the ischemic area and increase the cerebra infarct core [14]. Therefore, avoiding BP lowering treatment within the first 12 h might be reasonable in ischemic stroke patients. Unlike ischemic stroke, hematoma growth in ICH patients is not uncommon, and it occurs within the first few hours after onset of symptom [15]. Since hematoma growth is associated with poor outcome, interventions in an early period to limit active bleeding might be crucial for management of ICH [16]. Large randomized controlled trials have investigated the role of intensive BP reduction on hematoma expansion and functional outcome [8, 9]. The INTERACT2 trial was an international, multi-center, prospective, randomized controlled trial that included 2,794 spontaneous ICH patients with an initial SBP between 150 and 220 mm Hg within 6 h of ICH onset [8]. Participants were randomly assigned to receive either intensive treatment with a goal SBP of 110–139 mm Hg for 24 h or standard treatment with a goal SBP of 140–179 mm Hg. The percentage of deaths associated with ICH did not show significant differences between the intensive treatment group (61.4%) and the standard group (65.3%). The primary outcome did not show a significant difference between the two groups. The ATACH2 trial [9] compared intravenous nicardipine for intensive BP lowering (110–139 mmHg) versus standard treatment (140–179 mmHg) within 4.5 h of symptom onset in ICH patients, and no significant difference in death or disability at 90 days was observed (38.7 vs. 37.7%). Neither study reported a significant improvement in functional outcome between two groups.

In recent years, physicians have underscored the importance of controlling BP in their medical practice [17]. Although previous studies have focused on the effect of intensive versus standard blood pressure reduction on hematoma expansion and functional outcome [8, 9, 18, 19], few studies investigated the optimal timing for initiating BP reduction. In our study, we investigated the effect of early anti-hypertensive drug use (within 12 h after onset) on functional outcome in a cohort of continuously enrolled ICH patients. We found that early initiation of anti-hypertensive therapy was associated with improved functional outcome in acute ICH patients with SBP > 150 mmHg. Our study sheds light on the importance of timing of BP modulation on functional outcome. The time window for anti-hypertensive treatment is anticipated to be narrow because most hematoma growth occurs as early as the first few hours after ICH onset.

The optimal management after ICH remains undefined. The lack of improved outcomes in ICH patients may be linked to an excessive focus on intensive BP lowering as the sole therapeutic target. Considering the advanced age and presence of comorbidities in these patients, any potential treatment benefits may be offset by the development of medical complications during the acute phase. Therefore, improving functional outcome after ICH is likely to require a multi-faceted approach in early period after ICH [20]. The multi-center INTERACT3 (Intensive Care Bundle With Blood Pressure Reduction in Acute Cerebral Hemorrhage Trial) has reported the intervention of a goal-directed care bundle of intensive BP lowering, hyperglycemia control, antipyrexia treatment, and anticoagulation reversal, compared with usual standard of care, improves functional outcome in ICH patients [10].

Our study have some limitations. First, this study is a single-center study, which may have resulted in an underestimation of the association. Second, we were unable to provide detailed information about achieved BP and degree of BP lowering after early usage of anti-hypertensive drug, as that information was not readily accessible. Future randomized controlled trials are needed to elucidate the optimal timing and magnitude of BP reduction in patients with ICH.

## Conclusions

In conclusion, our study indicates that early anti-hypertensive treatment was associated with good outcome and functional independence among acute ICH patients with SBP > 150 mmHg at baseline. Our study sheds light on the importance of timing of BP modulation on functional outcome.

## Data Availability

Data available upon request from the corresponding author (QL). Ethical approval and consent to participants: The need for informed consent was waived by the Ethics Committee of the First Affiliated Hospital of Chongqing Medical University, because of the retrospective nature of the study.
